# An Investigation of Acoustic Back-Coupling in Human Phonation on a Synthetic Larynx Model

**DOI:** 10.3390/bioengineering10121343

**Published:** 2023-11-22

**Authors:** Christoph Näger, Stefan Kniesburges, Bogac Tur, Stefan Schoder, Stefan Becker

**Affiliations:** 1 Institute of Fluid Mechanics, Friedrich-Alexander-Universität Erlangen-Nürnberg, Cauerstraße 4, 91058 Erlangen, Germany; stefan.becker@fau.de; 2 Division of Phoniatrics and Pediatric Audiology, Department of Otorhinolaryngology, Head & Neck Surgery, University Hospital Erlangen, Medical School, Friedrich-Alexander-Universität Erlangen-Nürnberg, Waldstrasse 1, 91054 Erlangen, Germany; 3 Aeroacoustics and Vibroacoustics Group, Institute of Fundamentals and Theory in Electrical Engineering, Graz University of Technology, Inffeldgasse 16, 8010 Graz, Austria; stefan.schoder@tugraz.at

**Keywords:** human phonation, source–filter interaction, particle image velocimetry, synthetic larynx model, transmission line model, aeroacoustic source computation

## Abstract

In the human phonation process, acoustic standing waves in the vocal tract can influence the fluid flow through the glottis as well as vocal fold oscillation. To investigate the amount of acoustic back-coupling, the supraglottal flow field has been recorded via high-speed particle image velocimetry (PIV) in a synthetic larynx model for several configurations with different vocal tract lengths. Based on the obtained velocity fields, acoustic source terms were computed. Additionally, the sound radiation into the far field was recorded via microphone measurements and the vocal fold oscillation via high-speed camera recordings. The PIV measurements revealed that near a vocal tract resonance frequency *f_R_*, the vocal fold oscillation frequency *f_o_* (and therefore also the flow field’s fundamental frequency) jumps onto *f_R_*. This is accompanied by a substantial relative increase in aeroacoustic sound generation efficiency. Furthermore, the measurements show that *f_o_*-*f_R_*-coupling increases vocal efficiency, signal-to-noise ratio, harmonics-to-noise ratio and cepstral peak prominence. At the same time, the glottal volume flow needed for stable vocal fold oscillation decreases strongly. All of this results in an improved voice quality and phonation efficiency so that a person phonating with *f_o_*-*f_R_*-coupling can phonate longer and with better voice quality.

## 1. Introduction

The human voice is generated by a complex physiological process that is described by the fluid–structure–acoustic interaction (FSAI) between the tracheal fluid flow, structural vibration of laryngeal tissue (i.e., the vocal folds), and the sound generation and modulation in the larynx and vocal tract [1,2,3]. In this process, the two vocal folds are aerodynamically stimulated to vibrate by the airflow that arises from the lungs. In turn, this vibration leads to a modulation of the airflow, generating a pulsating jet flow in the supraglottal region, which is above the vocal folds [1].

Within this dynamic process of tissue–flow interaction, the basic sound of the human voice is generated by the highly complex 3D field of aeroacoustic sound sources which are produced by the turbulent jet flow in larynx [4]. Moreover, vibroacoustical sound generation also occurs by sound radiation from the vocal fold surface [5]. The generated basic sound is further filtered by the vocal tract and radiated through the mouth, exhibiting the typical spectral characteristics of the human voice composed of tonal harmonic components of the fundamental frequency and additional tonal components, called formants, originating from resonance effects in the vocal tract [6].

In early times, a linear behavior between sound source (vocal folds) and filter (vocal tract) was assumed within the linear source–filter theory, which excluded the influence of the acoustic filter signal back on the source [7]. However, this simplified representation turned out to be invalid, especially when the fundamental oscillation frequency of the vocal folds $f_{o}$ is close to a resonance frequency $f_{R}$ of the vocal tract [8]. Acoustic back-coupling has been studied using theoretical modeling (e.g., [9,10]), simulations (e.g., [11,12,13]), in vivo studies (e.g., [14,15]), and ex vivo and in vitro experiments (e.g., [16,17,18]). However, due to the complexity of the problem, most studies were restricted to metrics such as $f_{o}$-variation and the change in the subglottal oscillation threshold pressure. The direct acoustic–tissue or acoustic–flow interaction have not yet been investigated.

In this context, Particle Image Velocimetry (PIV) enables us to measure the unsteady flow field in the aeroacoustic source region to gain a deep insight into the FSAI process of phonation. This technique has already been applied successfully to study aerodynamic effects in synthetic as well as ex vivo larynx models that showed typical vocal fold vibrations similar to phonation. In this context, classical planar low-frequency PIV measurements allowed to study the basic features of the supra- and intraglottal aerodynamics [19,20,21,22]. Being still 2D, the emergence of high-speed PIV techniques made it then possible to directly analyze aeroacoustic source terms that were computed from time-resolved PIV data obtained directly in the source region above the vocal folds [5,23]. These data provided the distribution and dynamics of aeroacoustic sources based on state-of-the-art aeroacoustic analogies, such as the Lighthill analogy [24] or the Perturbed Convective Wave Equations [25]. With the rising availability of 3D tomographic PIV measurements, even first studies of volumetric parameters as the Maximum Flow Declination Rate were investigated using ex vivo canine models [26].

However, none of the studies described above have investigated the effects of supraglottal acoustics on the glottal aerodynamics and the aeroacoustic source field yet. Therefore, the present study provides highly resolved data of the entire process to analyze the complete FSAI between vocal tract acoustics and laryngeal aerodynamics. Based on high-speed PIV measurements in combination with aerodynamic and acoustic pressure data, as well as high-speed visualizations of the vocal fold dynamics in a synthetic larynx model [22], the influence of the resonance effects formed in the vocal tract on the supraglottal flow field and the vocal fold motion is studied. Different vocal tract models have been applied with an incremental increase in length. This length change produced acoustic properties of the vocal tract that shifted its resonance frequency down to the fundamental frequency of the vocal folds. This procedure enabled us to systematically study the relationship between laryngeal flow and supraglottal acoustics.

## 2. Materials and Methods

### 2.1. Basic Experimental Setup

**Figure 1:**
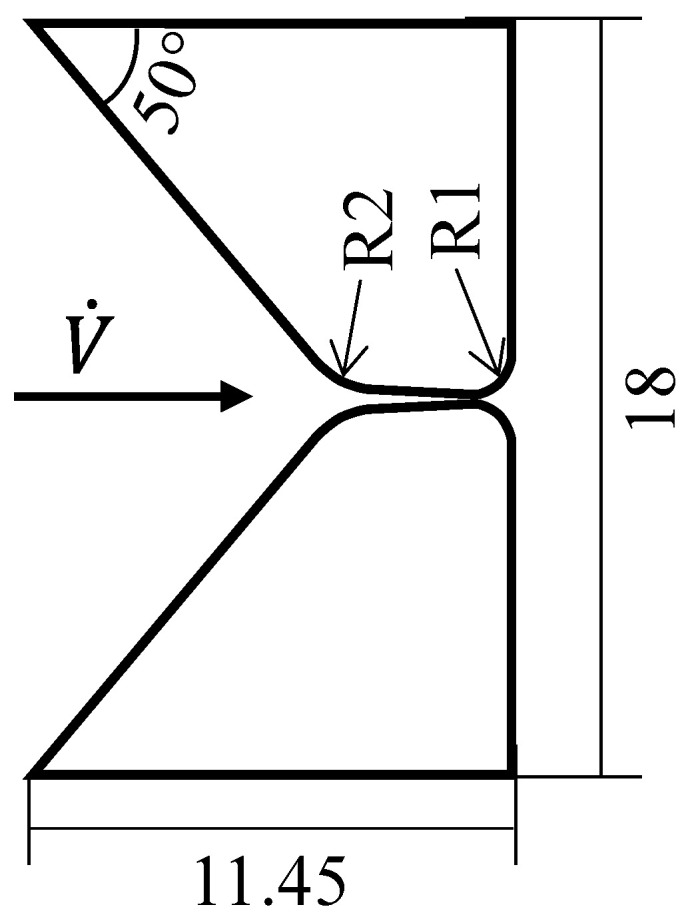
2D representation of the vocal fold model used, with its dimensions given in mm. The flow direction in the experiment is indicated.

**Figure 2:**
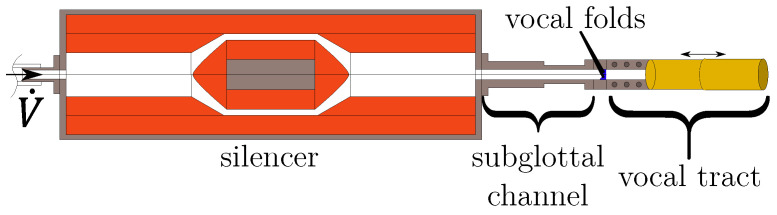
2D cut through the experimental setup. The vocal fold position is indicated between the vocal tract and the subglottal channel. A silencer is placed upstream to attenuate emerging sound in the inflow hose. The flow direction is from left to right.

Synthetic vocal folds were cast from a single layer of Ecoflex 00-30 silicone (Smooth-On, Macungie, PA, USA) with a static Young’s modulus of 4.4 kPa. Their shape was based on the M5 model [27,28], and is displayed in Figure 1. The vocal folds were glued into their mounting, positioned between the subglottal and supraglottal channels. In the prephonatory posturing of the vocal folds, the glottis was completely closed. The subglottal channel had a length of 210 mm and a rectangular cross-section of 18 mm × 15 mm, which is within the dimensional range found in vivo [1]. Furthermore, this length was chosen small enough to prevent the interaction of the vocal folds’ oscillation with the subglottal acoustic resonances (see the description in Lodermeyer et al. [21] based on the results by Zhang et al. [29]). The supraglottal channel had a rectangular section of 18 mm×15 mm and a length of 80 mm in the region directly downstream of the vocal folds. Attached to it, a circular cross-section tube with a diameter of 32 mm followed. An additional tube with a diameter of 34 mm made it possible to adjust the vocal tract length and thereby resonance frequency continuously via telescoping. This basic setup is shown schematically in Figure 2, and is based on the setup previously described by, e.g., Kniesburges et al. [22]. A mass flow generator with a supercritical valve [30] produced a constant volume flow $\dot{V}$ through the setup. Between the mass flow generator and the subglottal channel, a silencer was placed for conditioning the flow and attenuating sound propagation from the supply hose to the vocal fold position. Several measurements were performed for different vocal tract lengths in the interval L∈[200,800]mm to study the influence of the vocal tract acoustic resonance frequencies on the supraglottal flow field and vocal fold oscillation. The mass flow rate for each length was set to the corresponding minimum required for vocal fold oscillation with complete glottis closure.

### 2.2. Measurement Setup

Multiple measurement tasks were performed. The transglottal pressure was recorded via two pressure sensors: in the subglottal channel, a Kulite XCQ-093 sealed gauge pressure sensor (Kulite Semiconductor, Leonia, NJ, USA) was flush-mounted into the channel wall 50 mm upstream of the glottis. In the supraglottal channel, a Kulite XCS-093 open gauge pressure sensor (Kulite Semiconductor, Leonia, NJ, USA) was mounted the same way at a distance of 50 mm downstream of the glottis. The sound radiation from the vocal tract end was recorded in our anechoic chamber by a Brüel and Kjaer 4189-L-001 1/2″-microphone (Brüel and Kjaer, Nærum, Denmark) at a distance of 1 m perpendicular to the channel outlet. Microphone and wall pressure signals were sampled by a National Instruments PXIe 6356 multifunctional card (National Instruments, Austin, TX, USA) with a resolution of 16 bit and a sample rate of 44.1 kHz. The vocal fold movement was recorded using a Photron FASTCAM SA-X2 high-speed camera (Photron, Tokyo, Japan) at a frame rate of 10 kHz. From the microphone recordings, additional related parameters like the signal-to-noise ratio (SNR), harmonics-to-noise-ratio (HNR), as well as the cepstral peak prominence (CPP), were extracted using the Glottis Analysis Tools [31,32] (GAT; University Hospital Erlangen, Erlangen, Germany).

**Figure 3:**
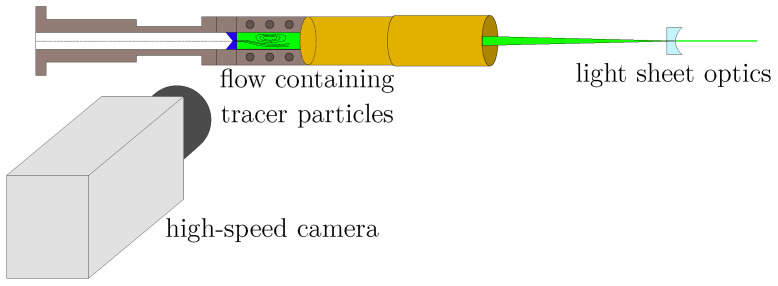
The setup for the PIV measurements. The flow was visualized using tracer particles and laser double pulses at a repetition rate of 2×5 kHz. The rectangular section of the vocal tract provided optical access through a glass window, allowing the flow to be recorded with a high-speed camera.

The planar flow velocity in the supraglottal region in the coronal plane midway along the vocal fold length was measured with a 2D-2C planar PIV setup. This setup is shown in Figure 3. The measurement region of interest was a rectangle with dimensions of 45 mm×18 mm and chosen similarly to the previous study by Lodermeyer et al. [5]. For seeding purposes, a PIVlight30 seeding generator (PIVtec GmbH, Göttingen, Germany) based on Laskin nozzles for atomization of the seeding fluid was applied. *PIVtec PIVfluid*, which is a propylene glycol mixture based on double de-ionized water and other components, was used as a seeding fluid, resulting in a mean particle diameter of 1.2 μm. The resulting Stokes number was St=0.033<0.1, yielding an acceptable flow tracing accuracy [33,34]. The particles were illuminated by a laser light sheet with a thickness of approximately 0.5 mm. The laser used in this study was a double-pulse, frequency-doubled Nd:YLF *Continuum Terra PIV* high-speed laser (Continuum, San Jose, CA, USA) with a wavelength of 527 nm and a repetition rate of 2×5 kHz. The offset between the two pulses was set to 4 μs, realized by an ILA synchronizer (ILA, Jülich, Germany). A *Vision Research Phantom v2511* high-speed camera (Vision Research Inc., Wayne, NJ, USA) in combination with a *Canon Macro Lens EF 180 mm Ultrasonic* lens (Canon, Tokyo, Japan) was applied to record the distribution of the illuminated seeding particles.

**Figure 4:**
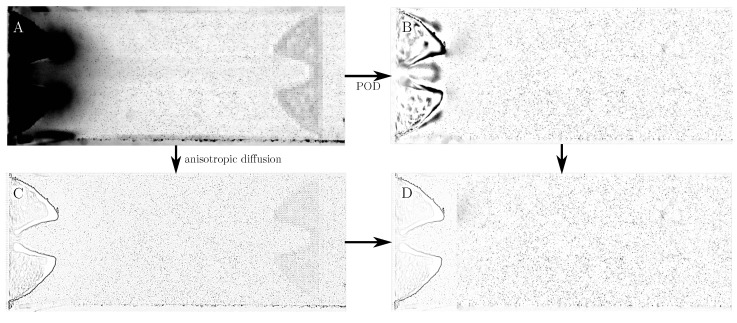
The different background removal techniques. The original images (**A**) are processed by POD (**B**) and anisotropic diffusion (**C**). The results are combined into one image (**D**), where the anisotropic diffusion image is used at the glottis, while the POD image is used in the other regions. The images were inverted to enhance visibility.

Two image pre-processing steps were applied to increase the signal-to-noise ratio in the recorded images. In the regions downstream of the vocal folds, a background removal via proper orthogonal decomposition (POD) proposed by Mendez et al. [35] was applied. This method is suited for removing background noise without moving walls, but showed poor background removal in the region close to the vocal folds. Therefore, a different approach was chosen for the region close to the glottis. Adatrao and Sciacchitano [36] proposed a background removal technique based on an anisotropic diffusion equation specifically for moving solid objects in PIV images. The results of both background removal techniques for one exemplary image are shown in Figure 4. It can be seen that the original image contains strong reflections at the vocal folds in the left part of the image. Also, sensor noise is visible in the rightmost quarter of the image. The POD-based background removal enables an almost complete removal of the sensor noise while removing most of the light reflections from the vocal folds. However, some artifacts are created around the vocal folds, masking the particle images, e.g., between the vocal folds. In contrast, the anisotropic diffusion-based background removal shows a sharp boundary around the vocal folds, improving the visibility of the particles between them. However, in this case, the sensor noise close to the right image boundary still remains present, albeit with reduced intensity. Therefore, to obtain the best result, the anisotropic diffusion approach is only used in the leftmost part of the images, while the POD-based approach is applied in the remaining part. Looking at Figure 4D, it appears as if there were less particles in the part close to the vocal folds visible than in the remaining image. This is a result of the light reflections from the vocal folds completely masking some particles in vicinity to the vocal folds. The PIV evaluation algorithm was still able to find enough particles in this region to obtain reliable velocity information, however.

Velocity vectors were extracted from the image pairs with the help of the commercial software PIVview2C 3.6.23 (PIVtec GmbH, Göttingen, Germany). For this purpose, a grid of 74×56 correlation windows with an overlap of 50% was defined in the region of interest, leading to a spatial resolution of Δx×Δy=0.62 mm×0.31 mm. Outliers were detected via the universal outlier detection by Westerweel and Scarano [37] and interpolated with the information from the surrounding velocity vectors.

### 2.3. Aeroacoustic Source Computation

One important aspect of understanding the human voice production process is to evaluate the aeroacoustic sources, e.g., Lighthill’s source term for low Mach number isentropic turbulent flows

(1) $$T\left(x,t\right)\approx \nabla \;\cdot \;\nabla \;\cdot \;\left(\rho_{0}uu\right)$$

with the velocities u measured via PIV and the ambient density $\rho_{0}$ [23]. This distributed source term is aggregated in a summed source strength based on Lighthill’s analogy [24] by neglecting the retarded time effects in this acoustically compact 2D region of interest (ROI)

(2) $$\phi \left(t\right)=\frac{1}{4\pi c_{0}^{2}\left(x_{1}-x_{0}\right)\left(y_{1}-y_{0}\right)}\int_{\left(x_{0},y_{0}\right)}^{\left(x_{1},y_{1}\right)}T\left(x,t\right)\frac{\pi ydxdy}{r}=\frac{1}{4c_{0}^{2}N_{x}N_{y}}\sum_{i}T\left(x_{i},t\right)|y_{i}|\;.$$

The coordinate locations $x_{0}$, $x_{1}$, $y_{0}$, $y_{1}$ are the bounding coordinates of the ROI, respectively, $c_{0}$ the isentropic speed of sound, *r* the direction of a virtual observer point at 1 m distance. It is assumed that the jet is rotationally symmetric around the rotation axis in *x*-direction, pointing in the flow direction and being centered in the middle of the vocal folds. From this equation, the root-mean-squared value is computed

(3) $$\Phi =\sqrt{\frac{1}{t_{1}-t_{0}}\int_{t_{0}}^{t_{1}}{\left(\phi \left(t\right)\right)}^{2}dt}\;,$$

being a measure of the ability to generate aerodynamic sound. The equation is applied to the measured 2D mid-section, where the velocity’s principal direction is recorded. In addition, the aerodynamic input energy is quantified by

(4) $$P=\Delta p+\frac{1}{2}\rho_{0}U^{2}\;,$$

using the subglottal pressure difference to the ambient pressure Δp. With the input energy, the efficiency of the aeroacoustic sound generation yields

(5) $$\eta =\frac{\Phi c_{0}^{2}\left(x_{1}-x_{0}\right)\left(y_{1}-y_{0}\right)}{P}\;.$$

### 2.4. Acoustic Characterization of the Vocal Tract

The acoustic properties of the vocal tract were determined using a transmission line model [38]. In this model, the acoustic pressure $p_{ac}$ and volume velocity $u_{ac}$ at the vocal tract input, i.e., the glottis, can be related to the same quantities at the output via chain matrix multiplication:

(6) $$\left(\begin{array}{c} p_{ac,out}\\ u_{ac,out} \end{array}\right)=K_{tract}\left(\begin{array}{c} p_{ac,in}\\ u_{ac,in} \end{array}\right)=\left(\begin{array}{cc} A_{tract} & B_{tract}\\ C_{tract} & D_{tract} \end{array}\right)\left(\begin{array}{c} p_{ac,in}\\ u_{ac,in} \end{array}\right)$$

Here, the 2×2-matrix $K_{tract}$ is built from chain multiplication of a series of 2×2-matrices $K_{i}$, each representing one part of the vocal tract with constant cross-section. These matrices $K_{i}$ were computed with the equations derived by Sondhi and Schroeter [38]. In the case of our simplified vocal tract, there are three different cross-sections present: the rectangular section right above the glottis, the circular section of the first tube and the circular section of the second tube. The vocal tract input impedance $Z_{in}=p_{ac,in}/u_{ac,in}$ can be obtained from Equation (6):

(7) $$Z_{in}=\frac{D_{tract}Z_{out}-B_{tract}}{A_{tract}-C_{tract}Z_{out}}$$

The maxima of the frequency-dependent $Z_{in}$ thereby correspond to the vocal tract resonance frequencies. The transmission line model was implemented following the description given by Story et al. [39]. As the vocal tract walls were fabricated from aluminum and glass, they were modeled as rigid walls. The radiation impedance at the open end was approximated as a vibrating piston in an infinite baffle [40].

## 3. Results and Discussion

### 3.1. General Results

As already stated in Section 2.1, the experiment’s volume flow rate $\dot{V}$ was set to the minimal flow rate necessary to induce oscillation with contact between the vocal folds. Table 1 lists the resulting $\dot{V}$ for all nine configurations measured. It can be seen that $\dot{V}$ stays roughly constant for L≤340 mm and decreases monotonically for larger *L*. The same behavior can be observed in the transglottal pressure $P_{trans}=P_{sub}-P_{supra}$, where $P_{sub}$ and $P_{supra}$ are the mean pressure values measured by the pressure probes in the sub- and supraglottal channel, respectively. The decrease in $\dot{V}$ with increasing *L* is similar to what Fulcher et al. [41] found, where they used an analytical surface wave model in combination with validation experiments to predict the phonation threshold pressure as a function of the vocal tract length. They related the decreased threshold pressure to an increase in vocal tract inertance due to the increased length.

**Figure 5:**
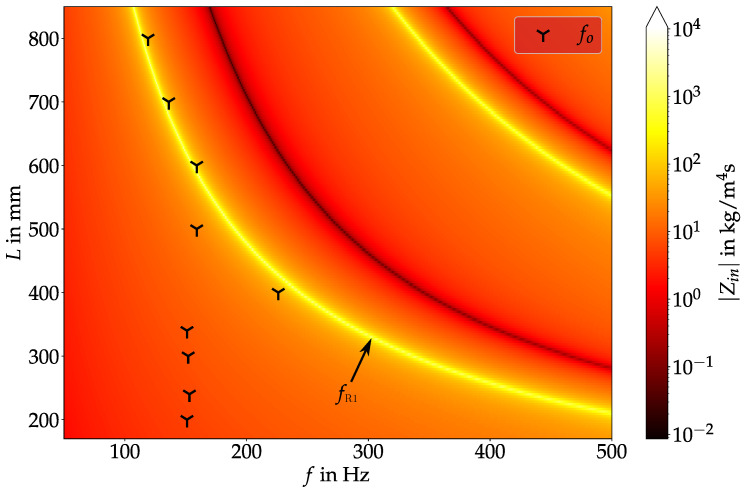
The vocal tract input impedance $Z_{in}$ (computed via transmission line model) shown as color map is a function of frequency *f* and vocal tract length *L*. The location of the vocal tract resonance frequency $f_{R1}$ shows as a bright yellow line in the plot. Superimposed are the oscillation frequencies $f_{o}$ at the individual measurements with different lengths of the supraglottal channel.

The oscillation frequency of the vocal folds $f_{o}$, as extracted via discrete Fourier transformation from the PIV-measurements also shows a stationary behavior in the range L≤340 mm. It stays within the range $150\;\mathrm{Hz}<f_{o}<153\;\mathrm{Hz}$ for these lengths. At L=400 mm, $f_{o}$ jumps to 225.7 Hz, which is 1.5-fold of 150.5 Hz. Increasing the length further leads to a jump back to ∼150Hz and then a decrease in $f_{o}$ down to 119.0 Hz. An explanation for this behavior can be found looking at the relationship between $f_{o}$ and the first vocal tract resonance frequency $f_{R1}$ as computed via the transmission line model. For this purpose, Figure 5 shows the vocal tract input impedance $Z_{in}$ as a function of the frequency *f* and *L*. The frequency of the maxima in $Z_{in}$ (indicated by the color yellow) correspond to the vocal tract resonance frequencies $f_{Ri}$. As expected, $f_{Ri}$ decrease with an increase in *L*. On top of the contour of $Z_{in}$, the values of $f_{o}$ for the different chosen vocal tract lengths from Table 1 are displayed.

**Figure 6:**
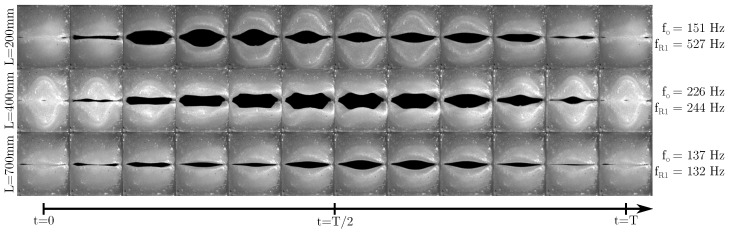
High-speed-camera recordings of the vocal fold oscillation for the three different vocal tract lengths of L=200 mm, L=400 mm and L=700 mm, respectively. Every row shows the recording for one length over one oscillation period *T*. Due to the different $f_{o}$-values, the actual time steps between two images are different in each row. The values for $f_{o}$ and $f_{R1}$ for all three cases are displayed to the right of their respective image series.

Here, it can be seen that for L<400 mm and L=500 mm, $f_{o}$ and $f_{R1}$ are not in vicinity to each other. Therefore, $f_{o}$ is approximately constant in this region, with the exception of small variations due to small changes in the experimental conditions as, e.g., a slight variation in $\dot{V}$. For L≥600 mm, $f_{R1}$ starts falling below 160 Hz and therefore lies in vicinity of the “uninfluenced” value of $f_{o}$. This leads to a decrease in $f_{o}$ with further increasing length in this length range. As a consequence, $f_{o}$ “jumps” onto $f_{R1}$ in this range and the vocal fold oscillation is coupled to the acoustic standing wave in the vocal tract. This is in accordance to the experimental data observed by Migimatsu et al. [18] in their experimental study with the M5 model. In their work, a much larger increase in *L* up to ∼1 m led to an $f_{o}$-jump back to the original uninfluenced value due to $f_{R1}$ being not in the vicinity of the uninfluenced $f_{o}$ anymore, leading to the domination of the vocal folds’ natural mechanical eigenmode. With our experimental setup, this could also be expected; however, this was not investigated. Zhang et al. also observed a locking of $f_{o}$ onto the supraglottal resonances [16]. Similarly, Zhang et al. furthermore showed, that also a locking of $f_{o}$ onto the resonance frequencies of the subglottal channel can occur, when they studied the influence of the subglottal resonances onto the vocal fold oscillation [16,29]. A special behavior happens at L=400 mm. Here, despite the uninfluenced $f_{o}$ lying still considerably below $f_{R1}$, a $f_{o}$ jump close to $f_{R1}$ takes place. As the oscillation at L=500 mm falls back to similar $f_{o}$ as at the smaller *L*, this gives the indication that there is a different behavior present at L=400 mm that occurs due to a special combination of the eigenmodes of the vocal folds and the acoustic resonance frequency of the vocal tract. To visualize this, high-speed camera videos have been recorded for three different *L*: 200 mm, 400 mm and 700 mm, respectively. These lengths were chosen, as they are representative of the three different states of vocal fold oscillation we were able to identify: independent $f_{o}$ and $f_{R1}$, a jump of $f_{o}$ to a higher frequency, and a shift of $f_{o}$ to lower frequencies. The relationship between $f_{o}$ and $f_{R1}$ for these lengths can be seen in Figure 5. Snapshots of one oscillation time period *T* for each of the chosen lengths are shown in Figure 6. The top row here corresponds to the baseline case, where $f_{o}\ll f_{R1}$. In this case, the vocal folds oscillate with a clearly visible convergent-divergent transglottal angle devolution, with a convergent glottal duct shape in the opening-phase and a divergent duct shape in the closing-phase. Similarly, at L=700 mm, the same behavior can be seen, albeit with a smaller opening area due to the reduced $\dot{V}$ in this case. In contrast, the oscillation at L=400 mm looks considerably different compared to the other two cases. Here, the change between convergent and divergent shape change in the glottal duct does not directly correlate with opening and closing motion of the vocal folds as described by Titze [42] for aerodynamically driven vocal folds. Therefore, combined with the $f_{o}$ jump to the vocal tract resonance frequency and a completely changed oscillatory behavior, this suggests that there is some kind of acoustic coupled motion of the vocal folds present in this case. As this only happens at L=400 mm, it is reasonable to assume that an eigenmode of the vocal fold model at a frequency of about 225 Hz is present in this case, that is excited by the acoustic standing wave of the vocal tract. This eigenmode is in the standard case not dominant compared to the 150 Hz-mode and therefore not visible with the other vocal tract lengths.

### 3.2. Supraglottal Aerodynamics

**Figure 7:**
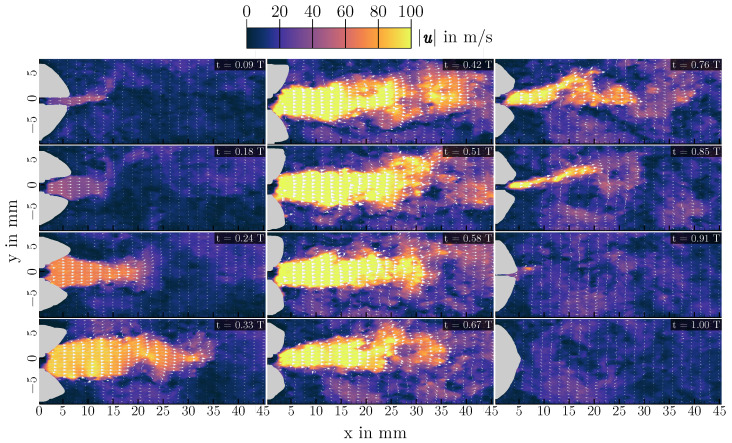
Instantaneous flow fields for 12 different time steps at a vocal tract length of L=200 mm. The time steps of the snapshots are shown in their respective top right corner.

**Figure 8:**
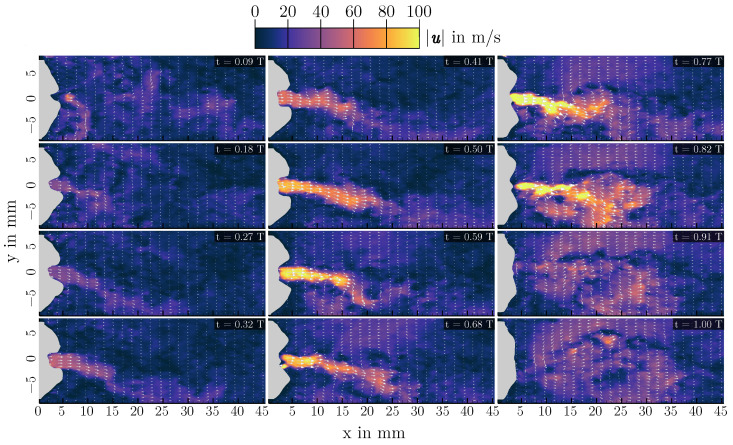
Instantaneous flow fields for 12 different time steps at a vocal tract length of L=400 mm. The time steps of the snapshots are shown in their respective top right corner.

**Figure 9:**
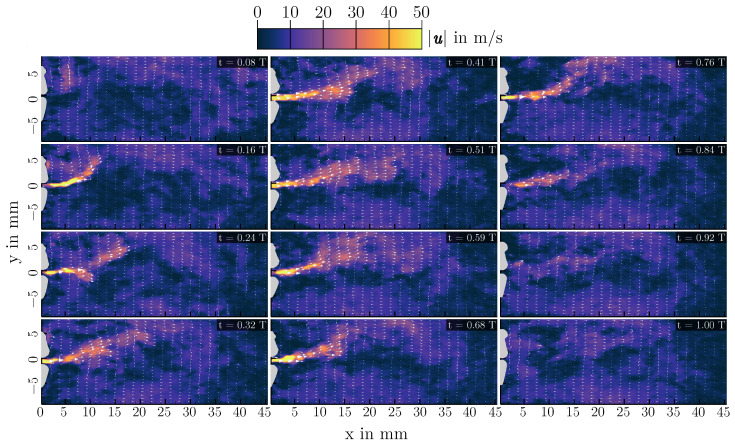
Instantaneous flow fields for 12 different time steps at a vocal tract length of L=700 mm. The time steps of the snapshots are shown in their respective top right corner. Note the changed color map limits compared to Figure 7 and Figure 8 for improved visibility.

**Figure 10:**
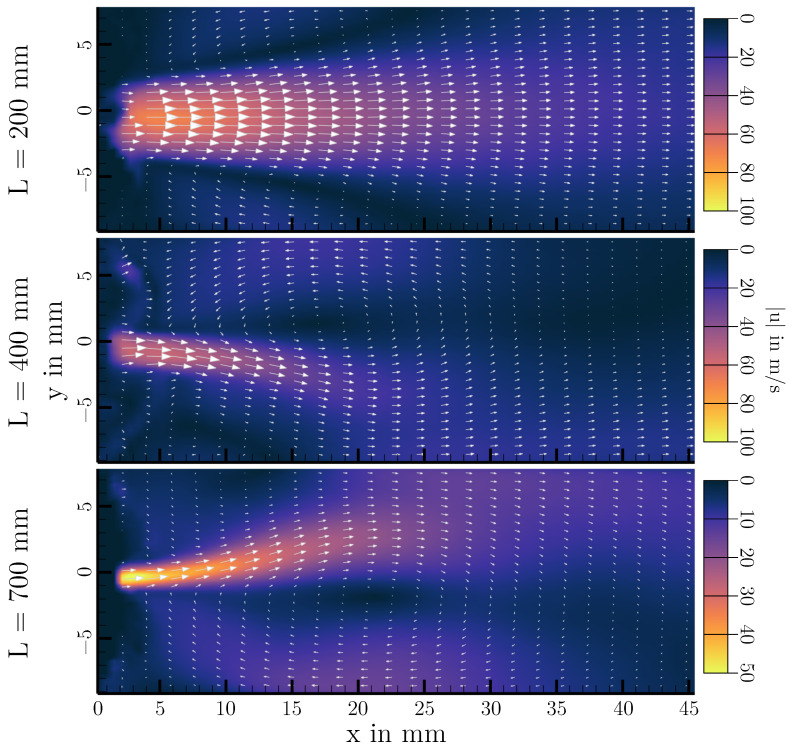
Mean velocity profiles for 200 mm, 400 mm and 700 mm.

PIV measurements have been performed for all configurations of Table 1. Again, the three cases of L=200 mm,L=400 mm and L=700 mm are analyzed in more detail as they are representative of the different possible oscillatory behaviors of the vocal folds. Velocity fields of one oscillation cycle for each length are shown in Figure 7, Figure 8 and Figure 9. Additionally, mean velocity fields for all three configurations are displayed in Figure 10. Looking at Figure 7, one can see that the basic characteristic of the flow is an oscillating jet synchronized to the opening and closing of the vocal folds (displayed in gray on top of the velocity contours). The jet is deflected during the closing phase to the top vocal tract wall, forming a large vortex in the supraglottal channel in the closed phase of the vocal folds. Depending on the cycle, also deflection of the jet downwards to the lower vocal tract wall can happen, leading to a vortex in the closed phase that is rotating in the other direction. This deflection and vortex formation is well known in human phonation, and has been studied extensively in the past (e.g., [21,22,43,44,45,46]). If there is approximately 50% of the cycles having an upwards deflection and 50% with a downwards deflection, this leads to a rather symmetric averaged velocity profile, as it can be seen in Figure 10 (top).

Looking at Figure 8, it appears that the basic characteristics of the flow are unchanged from L=200 mm to L=400 mm. There is still an oscillating jet flow, which is deflected to one of the vocal tract walls, leading to a large supraglottal vortex occurring during the closing and closed phase. Qualitatively, the acoustic driving of the vocal folds therefore does not appear to change the flow field in the middle plane of the vocal tract significantly. From an aerodynamic point of view, there are some changes, however related to the elongation of the vocal tract. In this case, the supraglottal jet is always deflected towards the lower vocal tract wall, leading to an asymmetry in the mean velocity field shown in Figure 10 (middle). Here, the supraglottal vortex is stabilized by the longer vocal tract. With a shorter vocal tract, the vortex is convected out of the vocal tract by the starting jet in the opening phase of the vocal folds. This leads to a new flow situation, where the jet deflection direction can be changed from one oscillation cycle to the next one. With a longer vocal tract, the vortex is just convected downstream inside the channel, thereby interacting with the jet starting from the vocal folds and deflecting it towards its side of positive x-velocities. Therefore, the direction of jet deflection in this case is dependent on the initial deflection at the beginning of the phonation. Kniesburges et al. observed a similar behavior when changing the supraglottal channel height (y-direction) [22]. Here, an increase in the channel height also led to a stabilized supraglottal vortex that interacted with the glottal jet flow. The jet deflection direction can also change from one phonation process to the next, as it can be seen when comparing the mean velocity profiles of L=400 mm and L=700 mm in Figure 10.

In the L=700 mm case, the jet is deflected upwards instead of downwards, also visible in the instationary velocity fields of Figure 9. Comparing the three configurations shown, the differences in the velocity magnitudes are notable, resulting from the different volume flow rates needed for vocal fold oscillation with contact. Generally, the peak flow velocities in this setup are higher than what is found in vivo [1], resulting from the large mean transglottal pressure needed for the single-layer synthetic vocal folds to oscillate with contact.

**Figure 11:**
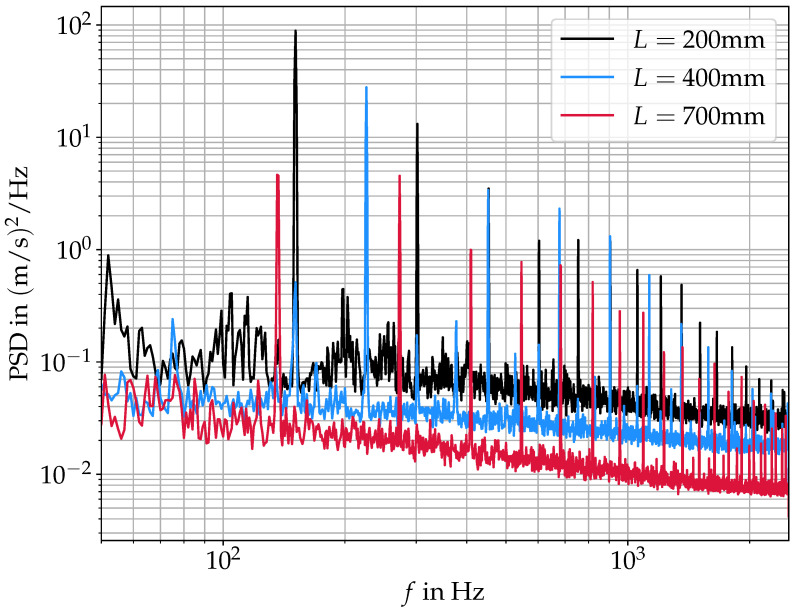
Averaged power spectral density of the flow field obtained by PIV for the three different vocal tract lengths of L=200 mm, L=400 mm and L=700 mm, respectively.

More quantitative differences between the three cases can be found by looking at the velocity fields in the frequency domain. Figure 11 shows the power spectral density (PSD) of the velocity magnitude averaged over the whole domain. All three spectra show the same qualitative trend of a general noise level decreasing with increasing frequency and strong harmonic peaks at their respective $f_{o}$ and higher harmonics. The $f_{o}$-shift according to the acoustic resonances as shown in Figure 5 and Table 1 is also observable here. Generally, the decreased velocity magnitudes with increasing *L* lead to a lower harmonic intensity as well as a lower noise level in the spectra. In the case of L=400 mm, there are also sub-harmonic peaks visible at $1/3f_{o}$, $2/3f_{o}$, $4/3f_{o}$, $5/3f_{o}$, and so on (with a fundamental frequency of $f_{o}=225\;\mathrm{Hz})$. In this case, the mode at 225 Hz is the strongest, while the 150 Hz mode is still visible in the spectrum. As can be seen in the spectra, the subharmonic peak at $2/3f_{o}$ coincides perfectly with the $f_{o}$-peak at L=200 mm. Therefore, this 150 Hz mode, as well as the peak at ∼75 Hz, can be interpreted as subharmonic peaks. Similarly, Titze [8] found the occurrence of subharmonic peaks at crossings of $f_{o}$ and $f_{R1}$ in a computational model studying the interaction of supraglottal acoustics and vocal fold oscillation. Kniesburges et al. [22] also observed the appearance of subharmonic peaks in the supraglottal aerodynamic pressure, as well as far field acoustic pressure in a synthetic larynx model. They attributed the subharmonic peaks to small changes in the supraglottal jet location from one oscillation cycle to the next one due to the supraglottal vortex changing direction from cycle to cycle. This, however, is not the same mechanism as apparent in the present study; if the change in rotational direction of the supraglottal vortex was the reason for the subharmonic peaks in our spectra, they would need to occur, especially in the case of L=200 mm, as here we have a symmetric mean velocity field (see Figure 10), indicating a 50:50 distribution of upwards and downwards deflection. In the case of L=400 mm, the supraglottal vortex is more stable, resulting in a 100% downwards deflection of the jet. This suggests that the subharmonic peaks are not produced by the supraglottal jet location in our case.

### 3.3. Aeroacoustic Sources

**Figure 12:**
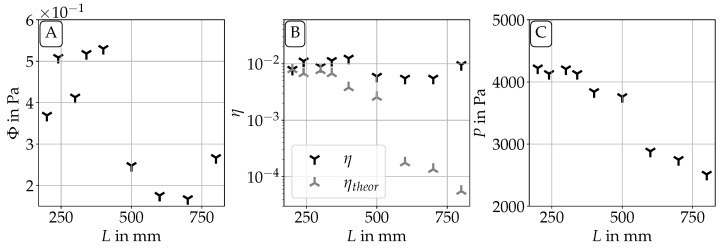
RMS aeroacoustic source level Φ (**A**), aeroacoustic efficiency η (**B**) and the total subglottal pressure *P* (**C**) of all vocal tract lengths *L* investigated.

To investigate the efficiency of the phonation process in the different cases, an aeroacoustic source term computation has been performed on the PIV measurements. For this, the Lighthill analogy was chosen. Root mean square (RMS) values Φ and an aeroacoustic efficiency η were computed as described by Equations (3) and (5). They are shown in Figure 12A and Figure 12B, respectively. It can be seen that Φ decreases with increasing length. This can be expected, as the aeroacoustic source intensity is dependent on the volume flow $\dot{V}$, which generally decreases with increasing length of the supraglottal channel. A similar trend can also be seen in the total subglottal pressure *P* shown in Figure 12C, which also shows a decrease with increasing length of the channel. Furthermore, the aeroacoustic efficiency η shows a slight decrease with increasing length up to a length of L=500 mm. For larger *L*, η is rather constant.

Lighthill [24,47] showed that the efficiency of sound generation in free turbulent flows without influence of solid walls in the flow domain scales with the fifth power of the Mach number. To compare our results to this scaling law, Figure 12B also shows a theoretical computation of the aeroacoustic efficiency $\eta_{theor}$, which makes use of this fifth power law. The case with L=200 mm is chosen as the baseline case. The proportionality constant of the power law is chosen so that $\eta =\eta_{theor}$ at L=200 mm. For the other cases, the value for $\eta_{theor}$ is then scaled with the fifth power of the corresponding bulk Mach number. For the channel lengths L≤340 mm, this law shows a reasonable agreement with the measurement data. It starts deviating from the data for larger *L*, and shows a strong difference for L≥600 mm. From Figure 5 we know that this is also the length region where $f_{o}$ is close to $f_{R1}$. This suggests that the acoustic resonance frequency of the vocal tract increases the aeroacoustic source intensity strongly by more than one order of magnitude. It also enhances the vocal fold oscillation, as the total subglottal pressure also needed for stable oscillation shows a strong drop by approximately 900 Pa. Overall, the aeroacoustic efficiency η is with approximately 1% higher than what Lighthill reported for free turbulent flows, which might be explainable by the assumptions we had to make due to the missing information in the third spatial dimension. The assumption of a rotational symmetry of the jet flow leads to a strong correlation in the circumferential direction, which increases the aeroacoustic efficiency. Another reason is that Lighthill did not take the existence of stationary or moving walls in vicinity to the flow field into account. Such walls are known to greatly increase the efficiency of sound production [48,49]. Despite these uncertainties, the η-values found are still useful for a relative comparison between the configurations.

### 3.4. Acoustic Radiation

**Figure 13:**
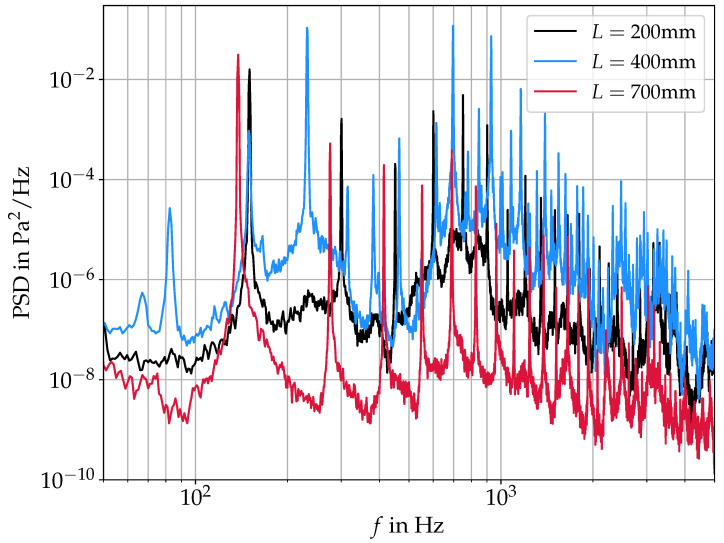
Acoustic spectra for the three different vocal tract lengths of L=200 mm, L=400 mm and L=700 mm, respectively.

**Figure 14:**
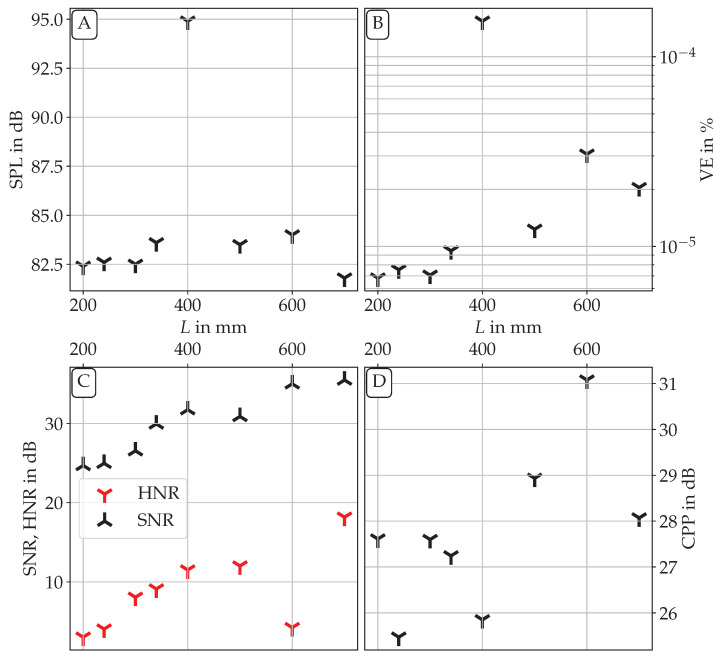
Sound pressure level (**A**), vocal efficiency (**B**), signal-to-noise ratio, harmonics to noise ratio (both **C**) and cepstral peak prominence (**D**) for all vocal tract lengths *L* investigated.

Figure 13 shows the acoustic spectra as measured by the microphone for the three main *L*. The difference in the $f_{o}$ values is apparent. Furthermore, for L=400 mm strong subharmonic peaks are visible. These subharmonic peaks can be directly related to the aerodynamic flow field, as they are at the same frequencies as in the aerodynamic spectrum of Figure 11. Also notable is the overall much lower noise level for L=700 mm. In contrast to the PIV-related spectra, the peak height at $f_{o}$ is, however, at a very similar level between the three measurements. This is the case due to the transfer function of the acoustic pressure of the vocal tract. As $f_{o}$ is very close to a resonance frequency of the vocal tract in the L=400 mm and L=700 mm cases, the transfer function of the vocal tract is very large for those frequencies, leading to an amplification compared to the other frequencies. To obtain a better insight into the changes in the acoustic radiation with changing *L*, several parameters have been computed from the microphone data. Figure 14A shows the overall sound pressure level (SPL) as a function of *L*. For most *L*, the SPL is in a range between 81.8dB and 84 dB. The one outlier is found for L=400 mm. Here, the SPL rises to almost 95 dB. In this case, two amplifying characteristics coincide: related to the large *P* and $\dot{V}$ values, the aeroacoustic source intensity and efficiency are very high (see Figure 12A,B). Additionally, $f_{o}$ is close to $f_{R1}$, leading to an amplification of the harmonic content in the acoustics. Therefore, a strong increase in the SPL can be expected. This also leads to a high vocal efficiency (VE, calculated following [50]), as seen in Figure 14B. Generally, the cases where $f_{o}$ is close to $f_{R1}$ show a higher vocal efficiency than the rest of the cases, while also showing higher values for the SNR [51] and the HNR [52], as computed by the GAT. For the case of L=400 mm, the difference in SNR and HNR compared to the other *L* are, however, much lower than in SPL and VE. This could be related to the strong subharmonic content in the acoustic spectrum, which leads to an erroneous noise content estimation. Also, the CPP [53,54] computed by GAT that is displayed in Figure 14D is very low for this case for the same reason. However, the CPP generally increases with increasing *L*, which indicates a decrease in noise, in contrast to tonal sound components. An outlier can be found for L=600 mm. Here, some high-intensity, low-frequency noise happened to disturb the acoustic signal at acquisition time, leading to a large low-frequency noise content. This led to a strong decrease in HNR for this length. As this noise increased the overall SPL, the vocal efficiency also supposedly increased here. Generally, the SNR values found for the cases without acoustic backcoupling (small *L*) lie in the range typical for a healthy voice [51]. With increasing backcoupling, the SNR increases even more, leading to an improved voice quality in these cases. The HNR values reported are in the range of values found for ex vivo studies in the literature [55,56]. They are, however, at the lower end of what is normally found in vivo [57,58,59]. In our case, this might be attributed to the high volume flow rates needed for the synthetic larynx model to oscillate, leading to increased turbulence broadband noise generation. The high volume flow rate is also responsible for the fact that the VE values are also rather low for all *L* compared to in vivo data [50].

## 4. Conclusions

Acoustic back-coupling in the human phonation process has been investigated using a synthetic larynx model and PIV measurements. The vocal tract length was changed systematically in the range L∈[200,800]mm to vary the relation between fundamental frequency of vocal fold oscillation $f_{o}$ and lowest resonance frequency of the supraglottal channel $f_{R1}$. The measurements showed that in the vicinity to each other, $f_{o}$ is tuned to $f_{R1}$. Decreasing $f_{R1}$ by increasing *L* led to a decrease in $f_{o}$ as well. Increasing $f_{R1}$ to a value higher than the uninfluenced $f_{o}$ generally did not increase $f_{o}$. One exception was the case for L=400 mm. Here, the vocal folds changed their vibration mode, triggered by the acoustic standing waves in the vocal tract having a frequency similar to the eigenfrequency of this mode. The acoustic resonance frequency of the vocal tract did not change the overall characteristics of the supraglottal aerodynamics. However, a changed vocal fold oscillation frequency naturally also led to the change in the dominant frequency in the pulsatile flow field. Looking at the aeroacoustic sources revealed that matching of $f_{o}$ and $f_{R1}$ resulted in a more than tenfold increase in aeroacoustic efficiency. This also led to an overall increased vocal efficiency, as well as increased SNR, HNR and CPP of the acoustic radiation. This indicates that, at this configuration, a person phonates with higher quality and efficiency. For the case of the professional female singing voice, where $f_{o}$ and $f_{R1}$ matching can occur at frequencies in the range of approx. 500Hz, this also means that the singer can phonate longer when $f_{o}$ and $f_{R1}$ match. This matching is also facilitated by the automatic tuning of $f_{o}$ to $f_{R1}$ we saw in vicinity.

The elongation of the vocal tract is a simplified approach to be able to study the phonation behavior for different $f_{o}$-$f_{R1}$ configurations. In reality, $f_{o}$-$f_{R1}$ matching only occurs when $f_{o}$ is close to lowest resonance frequency of the vocal tract being in the range of 500Hz, as stated above, which predominantly occurs in children and female singing voice [15]. To increase realism, in future works, more advanced synthetic vocal fold models could be used that show $f_{o}$ values in this range. Then, more anatomically realistic vocal tract shapes, e.g., from MRI-scans [60] could also be applied, leading to an overall more realistic configuration. Furthermore, the application of tomographic PIV or Lagrangian particle tracking methods could enhance the accuracy of aeroacoustic source term computation. In our case, the overall high aeroacoustic efficiency could be attributed to the rotational symmetry we assumed along the channel axis to obtain some information for the missing third spatial dimension. Tomographic methods would render this assumption unnecessary, improving the accuracy of our evaluation method.

## Figures and Tables

**Table 1:** The main measurement quantities of the PIV-measurements. *L* denotes the length of the supraglottal channel, $\dot{V}$ the flow rate, $P_{trans}$ the transglottal pressure difference, $f_{o}$ the oscillation frequency of the vocal folds and $f_{R1}$ the first resonance frequency of the vocal tract as computed via transmission line model.

*L* in mm	$\dot{V}$ in l/min	$P_{\mathrm{trans}}$ in Pa	$f_{o}$ in Hz	$f_{R1}$ in Hz
200	124	4208	151.3	527
240	120	4100	152.9	433
300	123	4188	151.3	337
340	120	4122	150.5	293
400	107	3791	225.7	244
500	99	3617	158.6	190
600	58	2659	158.6	156
700	55	2527	136.8	132
800	46	2266	119.0	114

## Data Availability

The data presented in this study are not publicly available due to ongoing research in this field.

## Abbreviations

The following abbreviations are used in this manuscript:

CPP	Cepstral Peak Prominence
FSAI	Fluid–Structure–Acoustic Interaction
GAT	Glottis Analysis Tools
HNR	Harmonics-to-Noise Ratio
PIV	Particle Image Velocimetry
POD	Proper Orthogonal Decomposition
PSD	Power Spectral Density
RMS	Root Mean Square
ROI	Region Of Interest
SNR	Signal-to-Noise Ratio
SPL	Sound Pressure Level
VE	Vocal Efficiency
